# Energy vs Charge Transfer in Manganese-Doped Lead Halide Perovskites

**DOI:** 10.1021/acsenergylett.1c00553

**Published:** 2021-04-23

**Authors:** Damiano Ricciarelli, Daniele Meggiolaro, Paola Belanzoni, Asma A. Alothman, Edoardo Mosconi, Filippo De Angelis

**Affiliations:** † Department of Chemistry, Biology and Biotechnology, University of Perugia, Via Elce di Sotto 8, Perugia 06123, Italy; ‡ Computational Laboratory for Hybrid/Organic Photovoltaics (CLHYO), Istituto CNR di Scienze e Tecnologie Chimiche “Giulio Natta” (CNR-SCITEC), Via Elce di Sotto 8, Perugia 06123, Italy; § Chemistry Department, College of Science, King Saud University, Riyadh 11451, Saudi Arabia; ∥ CompuNet, Istituto Italiano di Tecnologia, Via Morego 30, Genova 16163, Italy

## Abstract

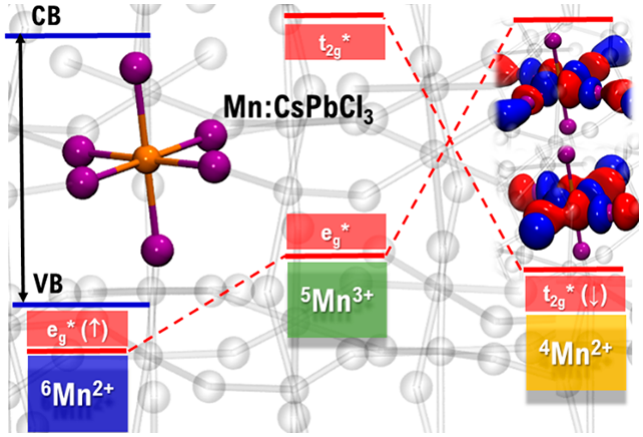

Mn-doped lead halide perovskites exhibit long-lived dopant luminescence and enhanced host excitonic quantum yield. The contention between energy and charge transfer in sensitizing dopant luminescence in Mn-doped perovskites is investigated by state-of-the-art DFT calculations on APbX_3_ perovskites (X = Cl, Br, and I). We quantitatively simulate the electronic structure of Mn-doped perovskites in various charge and spin states, providing a structural/mechanistic analysis of Mn sensitization as a function of the perovskite composition. Our analysis supports both energy- and charge-transfer mechanisms, with the latter probably preferred in Mn:CsPbCl_3_ due to small energy barriers and avoidance of spin and orbital restrictions. An essential factor determining the dopant luminescence quantum yield in the case of charge transfer is the energetics of intermediate oxidized species, while bandgap resonance can well explain energy transfer. Both aspects are mediated by perovskite host band edge energetics, which is tuned in turn by the nature of the halide X.

Doping semiconductors by transition metal ions has been widely proved to impart specific optical and magnetic properties to the semiconducting host. In particular, Mn^2+^ doping has been successfully implemented in binary semiconductor nanocrystals (NCs), such as CdSe, ZnS, ZnSe, and ZnO, with a direct impact on their stability and optoelectronic properties.^1−3^ As an example, doping CdSe NCs by Mn^2+^ substantially increases the excitonic photoluminescence (PL) lifetime. Another behavior which is also typically observed is the appearance of two PL features, i.e., the excitonic CdSe band at ∼2.4 eV and the typical ^4^T_1_→^6^A_1_ ligand field transition of the Mn^2+^ ion at ∼2 eV.^4^ Of technological relevance for optoelectronics and biomedical applications, the dopant emission is usually confined to a narrow energy range; thus, a variable Stokes shift can be realized by playing with both the size and the composition of the NC host. Furthermore, incorporation of manganese ions into the crystal lattice imparts magnetic properties to the host, clearly manifested at cryogenic temperatures.^5,6^

Metal halide perovskites (MHPs) are a game-changer class of materials in photovoltaics and optoelectronics.^7−16^ Besides their success in solar cells, perovskite NCs have shown outstanding optical properties in light-emitting diodes, with near unity emission quantum yield and wide color tuning.^17−24^ To further diversify the emission color gamut of MHP NCs, Mn^2+^ doping has been successfully implemented.^5,25,26^ Mn doping can be introduced in variable concentrations, covering a range up to ∼10%.^5,25−31^ Previous studies indicate incorporation of the transition metal in the bulk of the material rather than on the NC surface, with the transition metal located in a lead substitutional position within an octahedral coordination environment.^25,29,31,32^

Similar to what is observed in binary semiconductors, in MHPs a dual-color emission from the perovskite host exciton is observed, along with a broad Mn^2+^ (^4^T_1_→^6^A_1_) ligand field transition. The PL decay of the Mn emission is single exponential with a ∼ms lifetime,^27,30,32−34^ and it is moderately tunable by playing with the dopant content, varying from the yellow–orange region (2.14 eV) to the orange–red one (1.98 eV).^5,33,35,36^ Mn doping was also reported to enhance the MHP band gap PL quantum yield.^13,25,28,30,25,31,37−43^ The possible mechanism behind the dopant-induced PL quantum yield increase has been ascribed in some cases to dopant-to-material back transfer, although a plausible explanation is also defect passivation, e.g., Mn→Pb vacancy filling,^44^ which coherently suppresses material degradation and progressively stabilizes the host’s cohesive energy.^45^

Most notably, the Mn^2+^ luminescence quantum yield in perovskite NCs is strongly halide-dependent, with lead bromide perovskites showing a significantly less intense emission intensity compared to their lead chloride counterpart, while, to our knowledge, Mn^2+^ luminescence is not detected in lead iodide perovskites.^37−40,5,25,41−43^ This peculiar behavior raises questions about the interplay between dopant and host electronic energy levels and how this would affect the population of dopant states from initially populated host states. It is generally accepted that population of the ^4^T_1_ excited Mn^2+^ state occurs through energy transfer from the host exciton to the Mn dopant. In this framework, Liu et al. proposed that the more intense Mn^2+^ luminescence of lead chloride perovskites, compared to lead bromide and lead iodide compounds, can be ascribed to the progressively less resonant band gap of the perovskite host with the ligand field dopant transition.^5^ Pinchetti et al. pointed out the presence of a dopant sensitization process in CsPbCl_3_ occurring via an intermediate step which involves a long-lived shallow trap state mediating excitation of the Mn^2+^ center by the host band-edge excitons.^30^ Consistent with the proposed mechanism, an activation energy of ∼0.3 eV was measured for the sensitization process. Sun et al. confirmed the presence of an activation barrier for Mn^2+^ luminescence, though these authors found a smaller barrier of ∼0.1 eV.^29^ A similar effect was also detected by Zeng et al., with Mn^2+^ luminescence quantum yield decreasing with temperature.^46^

Despite available information, the nature of the mechanism responsible for the possible sensitization of the excited manganese ^4^T_1_ state remains elusive. Temperature-dependent Raman spectroscopy performed by Pradeep et al. on Mn:CsPb(Cl/Br)_3_ shows a progressive sharpening of the 132 cm^–1^ Pb–Br stretching phonon mode with increasing temperature, which is strongly coupled to Mn dopant modes.^41^ The presence of a transient Mn^3+^ species in Mn^2+^-doped II–IV semiconducting NCs was proposed by Gahlot et al.^47^ The presence of Mn^3+^ in perovskite samples was also invoked to rationalize the ferromagnetic coupling in MAPb_1–*x*_Mn_*x*_I_3_, proposed to arise from Mn^2+^–I^–^–Mn^3+^ motifs.^48^ Very recently, Babu et al. revealed the formation of a transient charge-transfer state in Mn:CsPbBr_3_, suggesting that a spin-allowed charge-transfer process (concurrent to energy transfer) related to Mn^3+^ formation is likely to occur.^49^

Motivated by the huge interest in Mn-doped MHPs and by the puzzling mechanism of Mn^2+^ sensitization, we here explore the structural and electronic properties of Mn-doped APbX_3_ perovskites (X = Cl, Br, and I) by state-of-the-art first-principles modeling studies based on hybrid density functional theory (DFT) and spin–orbit coupling (SOC) on fairly large perovskite models, Figure 1a. We accurately simulate the alignment of host/dopant energy levels in both Mn^2+^ and Mn^3+^ states considering different electronic and spin states and the associated structural rearrangement. Based on this data we provide an in-depth analysis on both the possible energy- and charge-transfer processes in Mn:APbX_3_ perovskites, highlighting points in favor of the former or the latter. In the framework of a hole (h) charge transfer to Mn^2+^ in CsPbCl_3_, we found a thermally activated mechanism with a barrier in fair agreement with that reported by Pinchetti et al.^30^ This suggests the same mechanism to be a major pathway in lead chloride perovskites, proposing Mn itself as a shallow trap mediating the sensitization of ^4^T_1_, later decaying to ^6^A_1_. We finally investigate the charge transfer/energy transfer dependency on different halides, extending our study to lead bromide and lead iodide perovskites.

**Figure 1:**
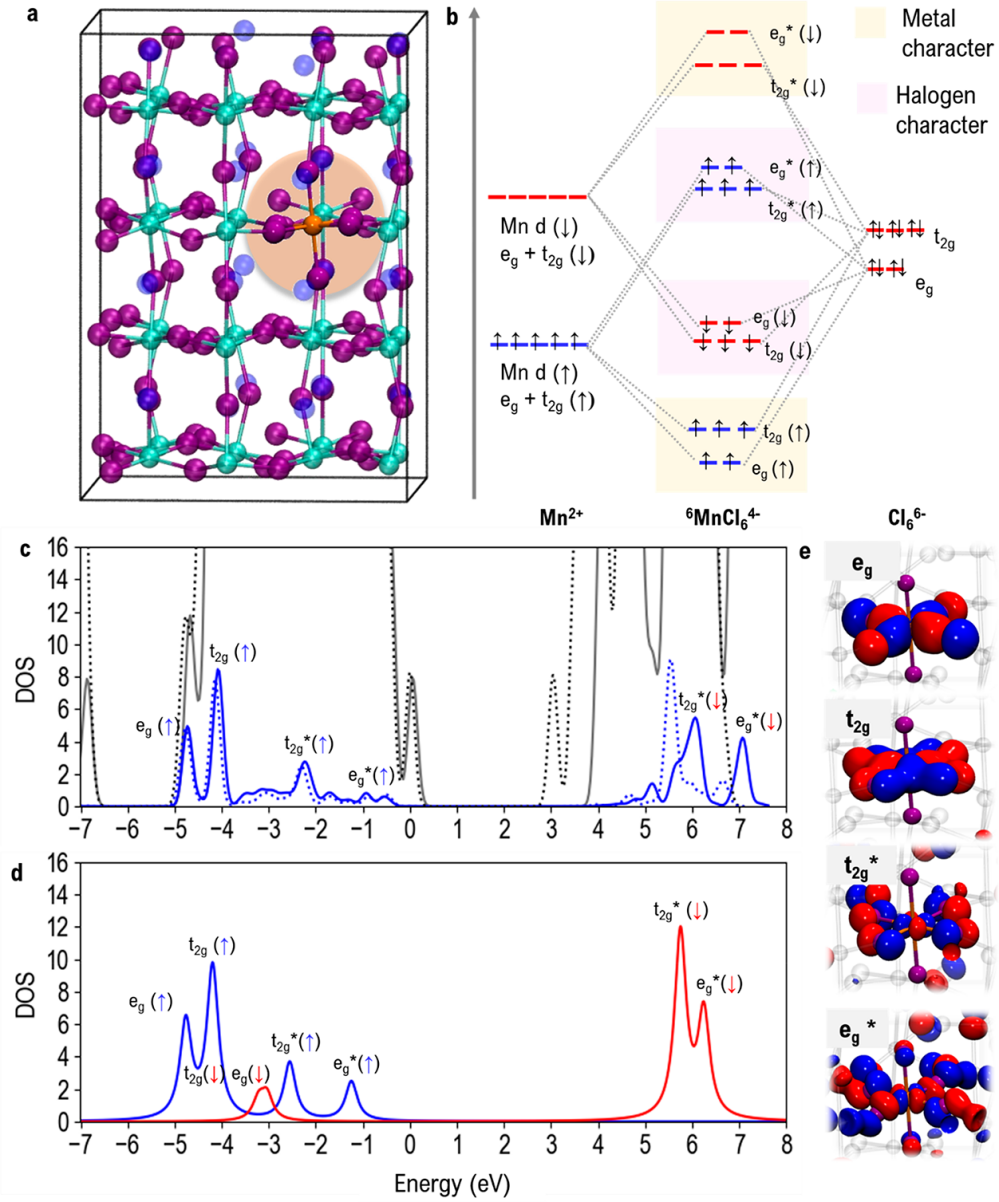
(a) Tetragonal 2×2×2 supercell (32 formula units) employed to model Mn:CsPbCl_3_. Orange, Mn; purple, Cl; cyan, Pb; and blue, Cs atoms. (b) Qualitative molecular orbital scheme for the complex with spin (↑) (blue) and spin (↓) (red) manifolds. (c) Total (gray) and manganese (blue) density of states (DOS) of ^6^Mn^2+^:CsPbCl_3_ computed at the PBE0 and PBE0-SOC levels (solid and dashed lines, respectively), with Mn molecular orbitals labeled. (d) Aligned manganese DOS for the ^6^MnCl_6_^4–^ model complex. (e) Isodensity plots of representative ^6^Mn^2+^:CsPbCl_3_ spin α single-particle states with e_g_, t_2g_, t_2g_*, and e_g_* character.

We model three key states of Mn-doped APbX_3_ perovskites. While the perovskite host has an even number of electrons, paired to form a singlet ground state, Mn doping introduces additional d-states of the dopant which can give rise to different electronic patterns, namely the sextet ground state of the Mn^2+^ ion with five Mn d-unpaired electrons (^6^A_1_, t_2g_^3^e_g_^2^, hereafter ^6^Mn^2+^), the quartet excited state (^4^T_1_, t_2g_^4^e_g_^1^, hereafter ^4^Mn^2+^) obtained by pairing two electrons from the high-spin sextet, and an oxidized state obtained by removing an electron from the sextet ground state, formally corresponding to a Mn^3+^ ion with a quintet ground state (t_2g_^3^e_g_^1^, hereafter ^5^Mn^3+^), which may account for hole trapping at the Mn^2+^ site.

We immediately face an issue with the coherent description of the electronic structure of the perovskite host—which requires the inclusion of SOC—and the interest in various local Mn spin multiplet states, which are not purely defined in the presence of SOC. In addition, we need to combine SOC with a hybrid functional, such as PBE0,^50,51^ for a correct description of the perovskite band edge energies and band gap. At the same time, a hybrid functional is required to correctly describe the energetics of the dopant d-shell orbitals and the structural relaxation associated with the various local Mn spin states.^52^ As a practical solution, by taking Mn:CsPbCl_3_ as a benchmark, we first calculate the electronic structure at the PBE0-SOC level, which provides results in quantitative agreement with the experiment (e.g., a host band gap of 3.04 eV, Figure 1c).^53^ Based on this analysis, we notice that unoccupied Mn states lie sufficiently above the host conduction band (CB) edge (>2 eV) that they are less likely to intrude in the gap when neglecting SOC (SOC basically downshifts the host CB states by ∼1 eV, while its effect on the valence band (VB) is negligible in this case, Figure 1c). We thus confidently employ PBE0 with no SOC for structural optimizations and for electronic structure analysis, checking the impact of SOC in selected cases.

The local electronic structure of ^6^Mn^2+^:CsPbCl_3_ shares many analogies with that typical of octahedral transition metal complexes with π-donor ligands,^54^ as schematized in Figure 1b, where a qualitative molecular orbital diagram for a ^6^MnCl_6_^4–^ model complex is reported. In the octahedral symmetry (*O*_*h*_), the Mn d orbitals labeled as e_g_ (d_*x*^2^–*y*^2^_, d_*z*^2^_) and t_2g_ (d_*xy*_, d_*xz*_, d_*yz*_), Figure 1b, interact with the halogen orbitals of the same symmetry, generating bonding molecular orbitals, i.e., t_2g_ and e_g_, and antibonding ones, i.e., t_2g_* and e_g_*, Figure 1e. The molecular orbitals with spins α and β are shown separately, referred to as (↑) and (↓) respectively, Figure 1b. The Mn contributions to the density of states (DOS) of Figure 1c for ^6^Mn^2+^:CsPbCl_3_ can be classified by comparison with the ones of ^6^MnCl_6_^4–^ in Figure 1d as following: e_g_ (↑) orbitals at ∼ –4.77 eV below the VB maximum, which are originated from the σ bonding interaction between the Mn d_*x*^2^–*y*^2^_, d_*z*^2^_ and Cl p orbitals, followed at ∼ –4.13 eV by t_2g_ (↑) orbitals, which originate from the π bonding interaction between the Mn d_*xy*_, d_*xz*_, d_*yz*_ and Cl p orbitals. Corresponding antibonding interactions can be found at ∼  −2.29 eV (t_2g_* (↑) orbitals) and at ∼ –0.70 eV (e_g_* (↑) states). Antibonding e_g_* and t_2g_* unoccupied (↓) states are found 1.63 eV above the SOC-calculated CB edge, Figure 1c. Notice that, in agreement with the model complex (Figure 1b–d), while the α bonding combinations show a predominant Mn character, α antibonding states have more halide character. The computed energy of occupied Mn (↑) 3d levels with respect to the material band edges appears in good accordance with that found for typical Mn-doped binary semiconductors like Cd_1–*x*_Mn_*x*_Te. The 3d^5^ levels of Mn(II)-doped Cd_1–*x*_Mn_*x*_Te are empirically located at 9.7 eV vs vacuum; thus, for a given work function (Wf, eV), subtract the Wf from 9.7 eV to find the binding energy (BE) of the Mn(II) levels below the Fermi level.^55,56^ In our case, with an experimental VB for CsPbCl_3_ located at −5.82 eV.^57^ we can expect the Mn levels to be 3–4 eV below the VB, in line with the calculated position of the t_2g_ (↑) and e_g_ (↑) states.

The ^6^Mn^2+^:CsPbCl_3_ ground state has an almost symmetric Mn–ligand coordination environment, Figure 2a, with average Mn–Cl distances of 2.57 Å, in good agreement with previous results reported by Ma et al.^58^ Significant structural differences in the MnCl_6_ coordination sphere are calculated for ^4^Mn^2+^:CsPbCl_3_ and ^5^Mn^3+^:CsPbCl_3_, Figure 2d–g, with compression (elongation) along the equatorial (axial) bond distances in ^4^Mn^2+^, Figure 2d, and average distances of 2.51 Å (2.63 Å). These structural differences can be understood in terms of the variation in the electronic structure associated with the different spin/charge states. ^4^Mn^2+^:CsPbCl_3_ is originated from ^6^Mn^2+^:CsPbCl_3_ by promotion of an electron from an e_g_* (↑) to an unoccupied t_2g_* (↓) state, initially lying within the perovskite host CB, Figure 2e,f. Although the octahedral symmetry is not retained, we continue to use octahedral labels for simplicity rather than *D*_4*h*_ labels; see Figure S1 in the Supporting Information.

**Figure 2:**
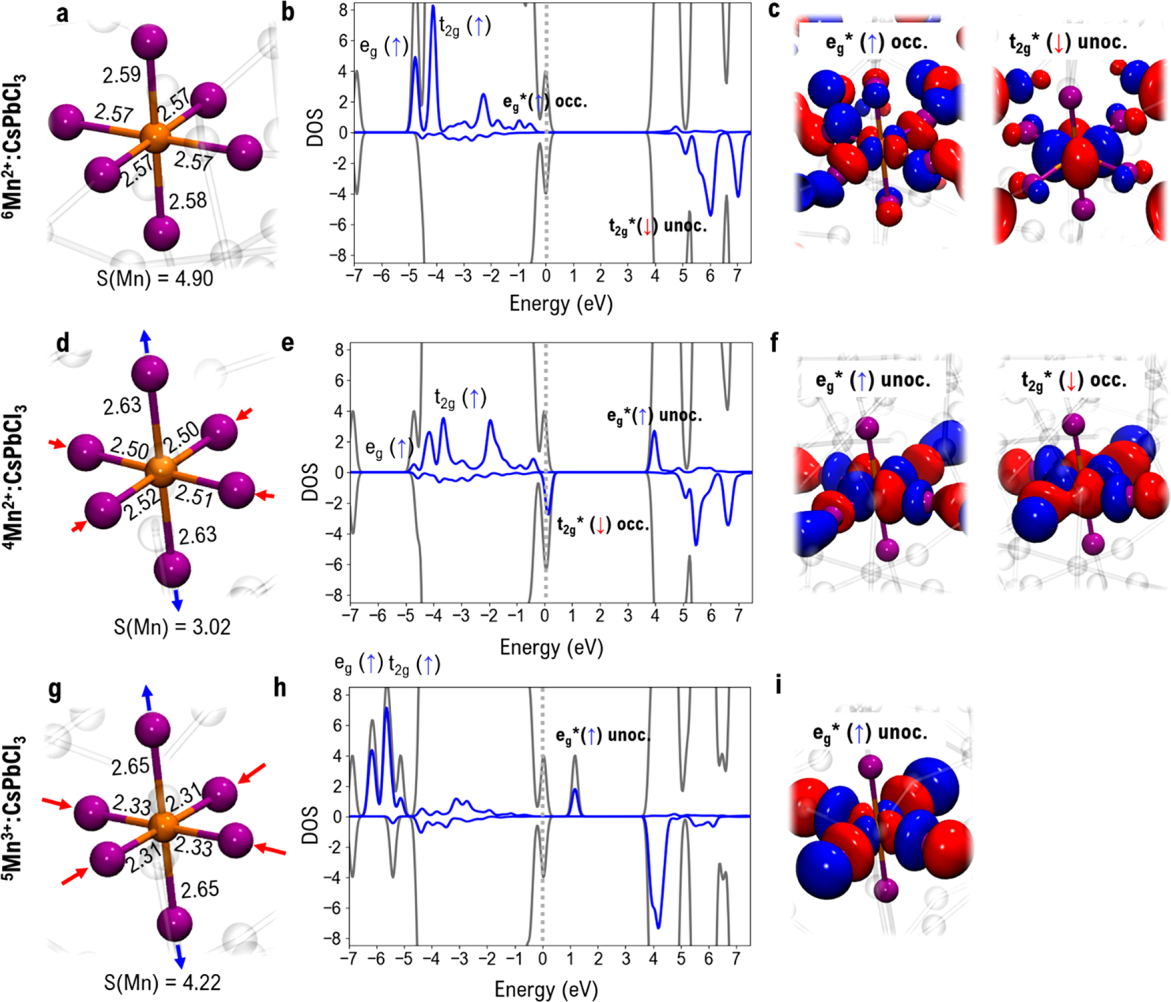
Structural properties, DOSs with Mn contributions, and key orbitals involved in spin/charge transitions for Mn:CsPbCl_3_. (a) ^6^Mn^2+^:CsPbCl_3_ first coordination sphere with distances in Å and spin moments from Mulliken population analysis. Orange spheres represent Mn atoms, purple ones chloride atoms; species outside the Mn first coordination shell are transparent. (b) Total (gray) and manganese (blue) DOS (with negative/positive spin differentiation) for ^6^Mn^2+^:CsPbCl_3_ computed at the PBE0 level of theory, with Mn active molecular orbitals labeled. The dashed vertical bar represents the Fermi level. (c) Isodensity plots of orbitals involved in spin/charge transitions for occupied e_g_* (↑) and unoccupied t_2g_* (↓) single-particle states of ^6^Mn^2+^:CsPbCl_3_. (d) ^4^Mn^2+^:CsPbCl_3_ first coordination sphere. (e) Total (gray) and manganese (blue) DOS for ^4^Mn^2+^:CsPbCl_3_. (f) Isodensity plot of orbitals involved in spin/charge transitions for unoccupied e_g_* (↑)and occupied t_2g_* (↓) single-particle states of ^4^Mn^2+^:CsPbCl_3_. (g) ^5^Mn^3+^:CsPbCl_3_ first coordination sphere. (h) Total (gray) and manganese (blue) DOS for ^5^Mn^3+^:CsPbCl_3_. (i) Isodensity plot of orbitals involved in spin/charge transitions for unoccupied e_g_* (↑) single-particle state of ^5^Mn^3+^:CsPbCl_3_.

The PBE0-calculated energy difference between the optimized ^4^Mn^2+^ and ^6^Mn^2+^ structures is 2.05 eV, in excellent agreement with the ^4^T_1_→^6^A_1_ PL recorded experimentally.^5,25,30,34,59,60^ A value of 1.48 eV was calculated by using the non-hybrid PBE functional, confirming the importance of the use of a hybrid functional for a quantitative assessment of Mn^2+^ electronic state energetics (see the related section in the Supporting Information to look for further GGA-hybrid comparative data).^52,61−63^ The charge hole left on the e_g_*(↑) state in ^4^Mn^2+^:CsPbCl_3_ is largely destabilized, now lying 0.01 eV above the CB, while the occupied t_2g_*(↓) state is strongly stabilized, now lying 0.13 eV above the VB; compare the DOS for the two states in Figure 2b–e and Figure S2 in the Supporting Information for additional analyses. A significant orbital reorganization occurs in parallel for e_g_ (↑) and t_2g_ (↑) states, whose density significantly decreases in ^4^Mn^2+^:CsPbCl_3_ compared to that in ^6^Mn^2+^:CsPbCl_3_, Figure 2b–e.

Upon removing one electron from the ^6^Mn^2+^:CsPbCl_3_ ground state, we find two structural minima with different energies and electronic structures; see Figure 2g and Figure S3a in the Supporting Information. The most stable oxidized species has a geometry similar to that of ^6^Mn^2+^:CsPbCl_3_, and inspection of the DOS (Figure S3b, Supporting Information) shows that it indeed corresponds to removal of one electron from the perovskite host VB, leading to a [^6^Mn^2+^/h^+^:CsPbCl_3_] electronic structure; i.e., the host rather than the dopant is oxidized. A secondary minimum corresponding to manganese oxidation, ^5^Mn^3+^:CsPbCl_3_, is calculated 0.18 eV above [^6^Mn^2+^/h^+^:CsPbCl_3_], which shows a significant equatorial compression (average Mn–Cl distance of 2.31 Å), Figure 2g, due to the typical Jahn–Teller effect of ^5^Mn^3+^ ions in weak ligand fields. The electronic structure of the ^5^Mn^3+^:CsPbCl_3_ DOS is reported in Figure 2h,i, which clearly shows hole localization in the e_g_* state, along with energy relaxation of bonding manganese states by ∼1.5 eV due to the increased manganese effective charge. It is interesting to notice that the [^6^Mn^2+^/h^+^:CsPbCl_3_] and ^5^Mn^3+^:CsPbCl_3_ minima are connected by an energy barrier of 0.33 eV, Figure 3, as determined by a linear transit calculation. On overall, while in ^4^Mn^2+^:CsPbCl_3_ a significant orbital reorganization occurs compared to ^6^Mn^2+^:CsPbCl_3_, in ^5^Mn^3+^:CsPbCl_3_ the distribution of t_2g_ (↑)(↓) and e_g_ (↑)(↓) states is almost unaltered, except for the aforementioned energy shift, Figure 2h. This behavior parallels what was found for the model complexes ^4^MnCl_6_^4–^ and ^5^MnCl_6_^3–^ (Figure S1, Supporting Information).

**Figure 3:**
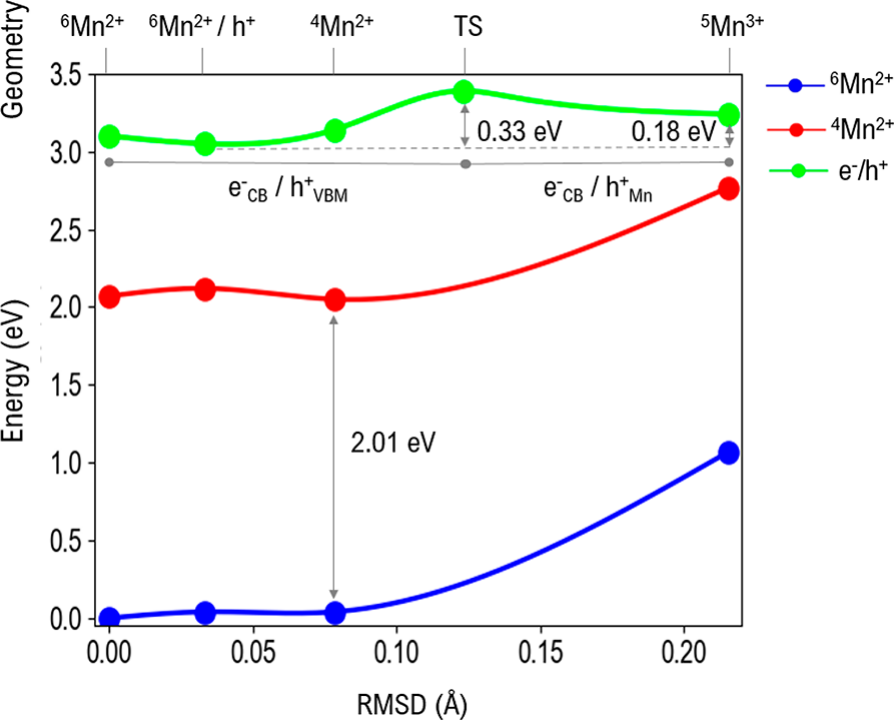
Configurational energy diagram of Mn:CsPbCl_3_. The energies of the sextet state (blue dots/lines), quartet state (red dots/lines), and electron/hole pair (green dots) are plotted against the root-mean-square deviation (RMSD) of the MnCl_6_ moiety in the perovskite. The reference is set as the sextet ground state. Larger values of RMSD correspond to MnCl_6_ equatorial bond compression and axial bond elongation.

In Figure 3 we propose a global spin/charge configurational diagram for Mn-doped CsPbCl_3_, with the three involved potential energy surfaces, i.e., ^6^Mn^2+^, ^4^Mn^2+^, and that obtained upon band gap excitation, lying 3.04 eV above the ground state. In this latter case we aim at describing the formation of an electron/hole pair, where the electron is delocalized into a host CB state, while the hole can be either VB-delocalized, as in [^6^Mn^2+^/h^+^:CsPbCl_3_], or trapped by manganese in ^5^Mn^3+^:CsPbCl_3_.

The potential energy surface of the three considered states is almost flat for low root-mean-square deviation (RMSD) values of MnCl_6_ (i.e., small structural distortions from the sextet ground-state geometry), while the total energy significantly increases when the Mn–Cl equatorial distances are compressed, moving to the ^5^Mn^3+^:CsPbCl_3_ minimum region, Figure 3. While the energies of the ^6^Mn^2+^:CsPbCl_3_ and ^4^Mn^2+^:CsPbCl_3_ states increase, that of the ^5^Mn^3+^:CsPbCl_3_ state goes through a transition state (TS) (0.33 eV above the [^6^Mn^2+^/h^+^:CsPbCl_3_] minimum) and then reaches a local minimum corresponding to hole trapping at the Mn center, which we calculate to be 0.18 eV higher than [^6^Mn^2+^/h^+^:CsPbCl_3_], Figure 3.

Upon band gap excitation of the CsPbCl_3_ perovskite host, there are two possible decay pathways involving the dopant:

(i) energy transfer from the host to the excited ^4^Mn^2+^:CsPbCl_3_ state;
(ii) hole trapping to form ^5^Mn^3+^:CsPbCl_3_, followed by non-radiative decay to ^4^Mn^2+^:CsPbCl_3_.

While the decay channel (i) requires negligible structural deformation, similar to band gap recombination, pathway (ii) requires overcoming an energy barrier associated with hole trapping with significant structural deformation. Previous literature typically ascribed population of the ^4^Mn^2+^ state to pathway (i), i.e., band gap exciton to manganese dopant energy transfer. A factor in favor of this mechanism is the small required structural rearrangement. An experimental finding which is also a point in favor of energy transfer is the mono-exponential Mn luminescence decay, suggesting the absence of intermediates.^27^ The different local Mn spin states could, however, hinder such a process; as a matter of fact, the direct ^6^T_1_→^4^T_1_ excitation has a negligible cross section due to the spin-forbidden nature of this process. Also, the significant orbital reorganization associated with the inner manganese shell discussed above could further inhibit such a process. Pathway (ii) requires a significant structural reorganization but no spin restriction, as ^5^Mn^3+^:CsPbCl_3_ can accept one electron in the t_2g_* state to form ^4^Mn^2+^:CsPbCl_3_, keeping the e_g_* orbital unoccupied. This mechanism of manganese ^4^T_1_ sensitization is associated with a calculated activation barrier of 0.33 eV, corresponding to the evolution of the transient ^5^Mn^3+^ species, which is in good agreement with the activation energy measured by Pinchetti et al.^30^ and consistent with the behavior of Mn:ZnSe NCs reported by Gahlot et al.^47^ The shallow trap in our case is represented by hole trapping at ^5^Mn^3+^:CsPbCl_3_. Coherently, Pradeep et al. reported temperature-dependent Raman spectra with sharpening of the 132 cm^–1^ peak (Mn/Pb–Br stretching mode) on Mn:CsPb(Cl/Br)_3_ NCs at temperatures >173 K, consistent with the occurrence of a significant Jahn–Teller distortion related to Mn oxidation.^41^

Since both energy- and charge-transfer processes are strongly dependent on the dopant/host relative energy levels, energetic overlap variations in the perovskite composition, in particular in terms of the halide nature, could afford significantly different electronic structures with different associated decay channels. To evaluate the electronic and structural changes occurring in different halide perovskites, we carried out a comparative study on CsPbCl_3_ and MAPbBr_3_/MAPbI_3_. The impact of A-site cations on the electronic structure is minimal; as a matter of fact, the ^4^T_1_→^6^A_1_ Mn luminescence is still observed,^64^ so we used pre-existing models for the bromide and iodide perovskites. Structural analyses indicate a progressive Mn–X bond increase with the series Mn–Cl ∼ 2.57 Å > Mn–Br ∼ 2.74 Å > Mn–I ∼ 2.98 Å, Table 1, in line with the increasing ionic radii trend.

**Table 1:** Averaged Mn–X Axial (*d*_Mn-X_(ax)) and Equatorial (*d*_Mn-X_(eq)) Distances (Å), Mn Spin from Mulliken Population Analysis (e), Band Gaps (Δ*E*_gap_, V), and ^4^T_1_→^6^A_1_ Spin Gaps (Δ*E*^4^ T_1_/^6^A_1_, eV)

**CsPbCl_3_**			
	Δ*E*_gap_ = 3.03		Δ*E*^4^T_1_/^6^A_1_ = 2.05
	*d*_Mn-X_(ax)	*d*_Mn-X_(eq)	Mn spin
^6^Mn^2+^	2.58	2.57	4.90
^4^Mn^2+^	2.63	2.51	3.02
^6^Mn^2+^/h^+^	2.59	2.56	4.90
TS	2.64	2.42	4.74
^5^Mn^3+^	2.65	2.32	4.22


**MAPbBr**_**3**_			
	Δ*E*_gap_ = 2.34		Δ*E*^4^T_1_/^6^A_1_ = 2.04
	*d*_Mn-X_(ax)	*d*_Mn-X_(eq)	Mn spin
^6^Mn^2+^	2.77	2.72	4.92
^4^Mn^2+^	2.83	2.65	3.07
^6^Mn^2+^/h^+^	2.77	2.73	4.92
TS	2.83	2.56	4.62
^5^Mn^3+^	2.85	2.52	4.48


**MAPbI**_**3**_			
	Δ*E*_gap_ = 1.80		Δ*E*^4^T_1_/^6^A_1_ = 1.99
	*d*_Mn-X_(ax)	*d*_Mn-X_(eq)	Mn spin
^6^Mn^2+^	3.04	2.95	4.92
^4^Mn^2+^	3.09	2.89	3.15
^6^Mn^2+^/h^+^	3.04	2.95	4.92
^5^Mn^3+^	3.09	2.72	4.59

We notice that our calculations retrieve the experimental trend of VB rising and band gap closing observed for the Cl → Br → I series, while the electronic structure of the Mn-doped systems is qualitatively similar along the halide series, although quantitative differences are found in the distribution of the bonding/antibonding states. Figure 4, panels a, c, and e, clearly show that the Mn–Cl bonding states, i.e., e_g_ and t_2g_, contain progressively smaller halide contributions when moving from Cl to I, in line with ^6^MnX_6_^4–^ models (Figure S4, Supporting Information). This could be ascribed to the reduced covalency in the Mn–X bond and to the reduced ligand field strength. The inner manganese states become harder because of the combined less covalent character of the chemical bond and weaker ligand field provided by the halides according to the I^–^ < Br^–^ < Cl^–^ trend.

**Figure 4:**
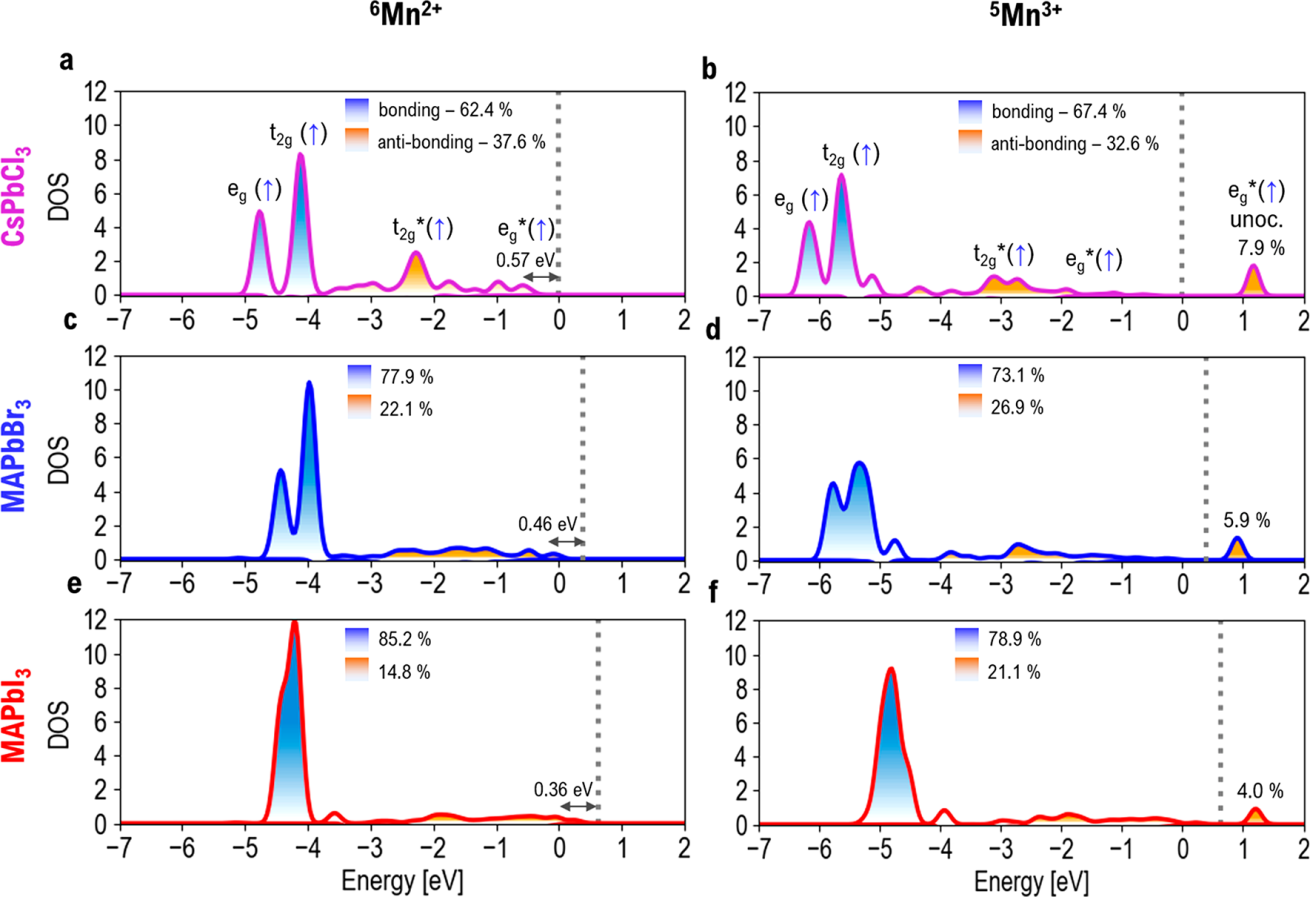
Manganese DOS contribution in ^6^Mn^2+^ and ^5^Mn^3+^ electronic states for Mn-doped CsPbCl_3_, MAPbBr_3_, and MAPbI_3_ perovskites. The manganese–halide bonding (antibonding) states are highlighted in blue (orange), and the percentage weight of each region compared to the total is also reported. The VB of CsPbCl_3_ is set to zero, while the energies of Mn-doped MAPbBr_3_ and MAPbI_3_ perovskites are aligned to that of Mn-doped CsPbCl_3_. The dashed lines represent the aligned VB edges.

In line with results on CsPbCl_3_, in the bromide and iodide perovskites, the ^4^Mn^2+^ structures present a slight equatorial compression and axial expansion; see Table 1. The energy of the quartet ^4^T_1_ vs ^6^A_1_ is ∼2 eV for the three perovskites, irrespective of the nature of the halide. These values well agree with the emission detected experimentally,^5,41,65^ confirming the insensitivity of the Mn-dopant emission energy to the host chemical structure. A significantly higher energy state is found in the case of Mn:MAPbI_3_, characterized by a Mn spin moment of 1.12 (see results in the Supporting Information), related to the reduced covalency and ligand strength of the Mn–X bond.

Similar to what has already been discussed for CsPbCl_3_, when a positive charge is allocated to the lead bromide or lead iodide perovskites, a global minimum is found where the perovskite host VB is oxidized, followed by a higher energy local minimum where Mn is oxidized, with subsequent equatorial (axial) compression (elongation), Figure 5g–j. In the case of bromide, the ^5^Mn^3+^ minimum is 0.34 eV above the ^6^Mn^2+^/h^+^ state, and it can be reached by overcoming a barrier of 0.35 eV, while in the case of the iodide, ^5^Mn^3+^ minimum is 0.40 eV above ^6^Mn^2+^/h^+^, Figure 5h–k.

**Figure 5:**
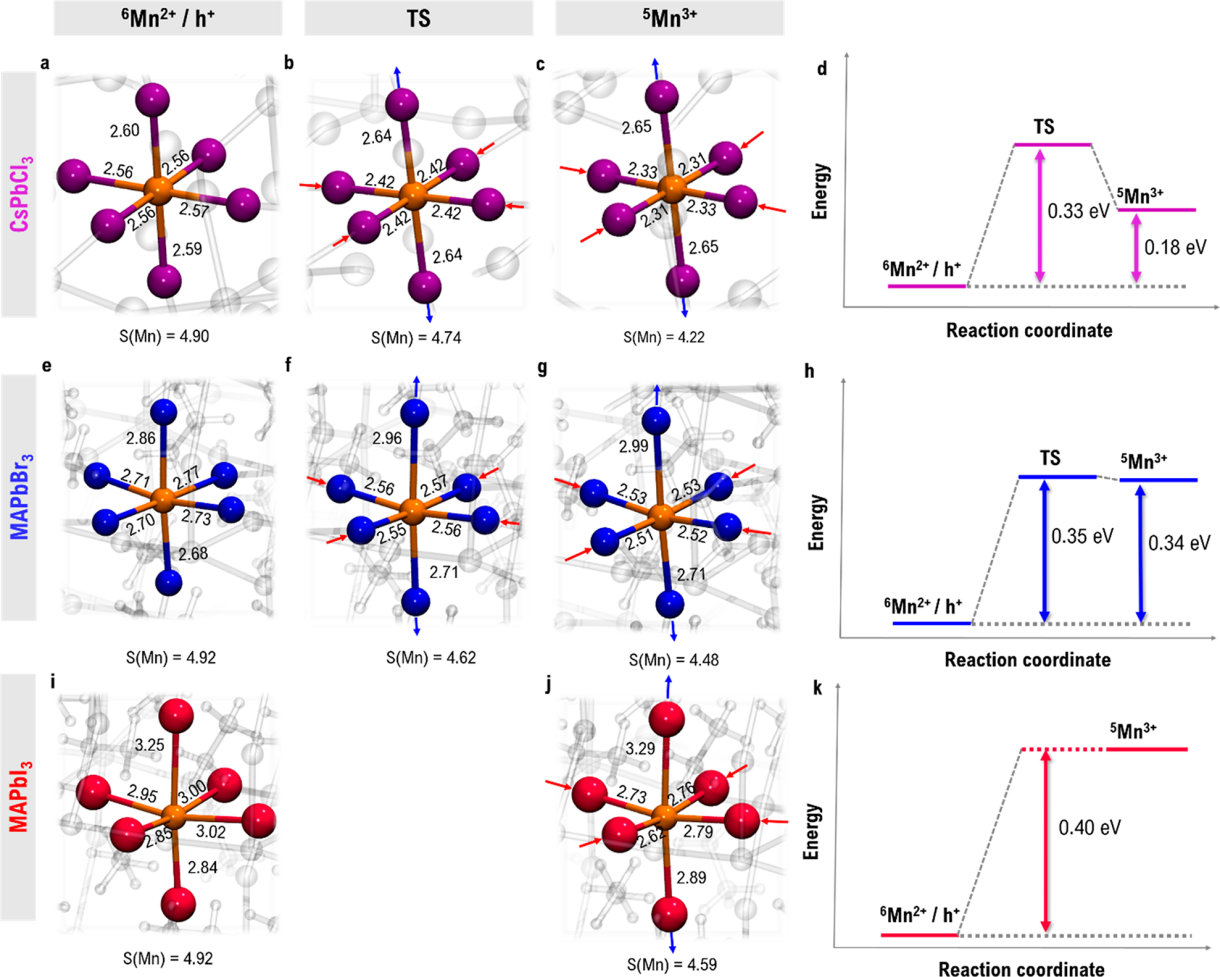
Mn coordination spheres and energy features of key species along the route of Mn oxidation in the quintet spin state: ^6^Mn^2+^/h^+^ with a free hole delocalized in the material VB, the transition state (TS) connecting ^6^Mn^2+^/h^+^ to ^5^Mn^3+^, and ^5^Mn^3+^ corresponding to the oxidized manganese. Mn spin moments are reported along with octahedron distances in Å. Orange spheres represent manganese atoms, purple ones chloride atoms, blue ones bromide atoms, and red ones iodide atoms; species outside the Mn first coordination shell are transparent.

The energy difference between ^5^Mn^3+^ and ^6^Mn^2+^/h^+^ increases when moving from Cl (0.18 eV) to Br (0.34 eV) and I (0.40 eV), highlighting a progressive thermodynamical destabilization of the oxidized form, although their kinetic barriers are similar. Considering the energy-transfer process of pathway (i), we should expect, as proposed by Liu et al.,^5^ to observe a band-gap-dependent Mn-dopant luminescence. Basically, to observe Mn PL, the host band gap should be larger than ∼2.0 eV, i.e., the energy of the (^4^T_1_→^6^A_1_) ligand field transition. The charge-transfer pathway (ii) is instead modulated by the different thermodynamic stability of the oxidized forms and the corresponding kinetic barriers, Figure 5. We predict that formation of ^5^Mn^3+^ requires similar activation energies in the three materials, which should induce a similar PL quantum yield temperature trend for the three halides. The reduced stability of ^5^Mn^3+^ in the order Cl > Br > I, however, indicates that the concentration of dopant luminescent centers is significantly reduced in the latter two cases, progressively hindering the dopant PL quantum yield.

To sum up, our accurate theoretical analysis has allowed us to quantify the dopant/host alignment of energy levels for a series of manganese-doped lead halide perovskites, identifying the key factors responsible for energy- vs charge-transfer population of the ^4^Mn^2+^ excited state. Our analysis supports both energy-transfer and charge-transfer mechanisms, with the latter probably preferred in Mn:CsPbCl_3_ due to avoidance of spin and orbital restrictions, though requiring a significant structural modification at the dopant site. Also in favor of a charge-transfer mechanism in this case is the calculated activation energy for manganese sensitization, agreeing with experimental data for the ^4^T_1_→^6^A_1_ luminescence. The reported quantitative comparison of the electronic structure of different halide perovskites may well rationalize the progressive disappearance of dopant luminescence on the basis of both energy-transfer and charge-transfer mechanisms. For the latter we found that an essential factor in determining the Mn quantum yield, in addition to the host band gap/Mn ^4^T_1_→^6^A_1_ resonance, is provided by the thermodynamics/kinetics of ^5^Mn^3+^ formation. The kinetics is similar, and this predicts PL temperature trends analogous to those of to Mn:CsPbCl_3_, but the Mn oxidized species is thermodynamically more stable in chloride than in bromide or iodide perovskites, implying a reduced concentration of Mn chromocenters in the latter two systems.

## Computational Details

Mn-doped perovskites CsPbCl_3_, MAPbBr_3_, and MAPbI_3_ were modeled in the 2×2×2 supercell of tetragonal APbX_3_. The dopant Mn was disposed in a substitutional position, in place of the metal lead, assuming a doping concentration of 3.12%. Equilibrium structures were found by relaxing ion positions in the supercell defects by using the PBE0 functional and fixing cell parameters to the experimental values: for CsPbCl_3_, *a* = *b* = 15.811 Å, *c* = 22.520 Å; for MAPbBr_3_, *a* = *b* = 16.690 Å, *c* = 23.604 Å; and for MAPbI_3_, *a* = *b* = 17.711 Å, *c* = 25.320 Å.^66,67^ Hybrid functional PBE0 calculations were performed by using the CP2K code,^68,69^ keeping the fraction of Fock exchange α at its original value (0.25). Calculations were carried out with Goedecker–Teter–Hutter pseudopotentials, six double-ζ polarized basis sets for the wave functions, and a cutoff of 500 Ry for the expansion of the electron density in plane waves.^70^ We used the auxiliary density matrix method with the cFIT auxiliary basis set to speed up the hybrid functional calculations. To simulate the different spin properties of our systems, the calculations were all performed with the local spin density (LSD) approximation by fixing the spin multiplicity to the respective value, i.e., 6 for ^6^Mn^2+^:APbX_3_, 4 for ^4^Mn^2+^:APbX_3_, and 5 for ^5^Mn^3+^:APbX_3_ and ^6^Mn^2+^+h^+^:APbX_3_. The atomic Mn spin moments of the minima were calculated by employing the Mulliken population analysis.^71^

Additional PBE0-SOC calculations for ^6^Mn^2+^:CsPbCl_3_/MAPbBr_3_/MAPbI_3_ were performed by means of single points on the PBE0 structures using a PBE0 hybrid functional^50,51^ together with SOC corrections.^72^ Norm-conserving pseudopotentials were used with a cutoff on the wave function of 40 Ry and a cutoff on the Fock grid of 80 Ry, sampling at the Γ point of the Brillouin zone. The fraction of exact exchange was kept to its original value of α = 0.25. To this aim we used the Quantum Espresso package.^73^

To compare energy values of positively charged systems, such as ^5^Mn^3+^ and ^6^Mn^2+^+h^+^, to those of the neutral ground state, we computed the respective redox level (0/+),^44^ making use of the Freysoldt approach for the finite size correction.^74^

The alignment of valence bands and Mn levels for different halides perovskites in Figure 4 was achieved using the tridimensional averaged Hartree potential.

Comparative molecular calculations on the model complexes MnX_6_^4–^/MnX_6_^3–^ were carried out by employing the Amsterdam Density Functional program (ADF) using the PBE0 functional,^50^ a Slater-type triple-zeta basis set augmented with two polarization functions (TZ2P), and a scalar Zero Order Regular Approximation to the Dirac Equation (ZORA) Hamiltonian.^75,76^ Molecular orbital diagrams were constructed through the symmetrized fragment orbital approach by dividing the complex in two fragments, i.e., Mn^2+^/Mn^3+^ and 6Cl^–^. We applied octahedral (*O*_*h*_) molecular symmetry for ^6^MnX_6_^4–^ and tetragonally distorted (*D*_4*h*_) symmetry for ^4^MnX_6_^4–^ and ^5^MnX_6_^3–^.^75^

## Supporting Information Available

Structures, DOS, and orbitals of Mn:APbX_3_ and MnX_4_^–^ (PDF)
