# Supplementary material for: Energy vs Charge Transfer in Manganese-Doped Lead Halide Perovskites
Source: ACS Energy Lett. 2021 Apr 23;6(5):1869–78. doi: 10.1021/acsenergylett.1c00553 (PMC8763376; doi:10.1021/acsenergylett.1c00553)
Supplement: Supplementary file 1 — nz1c00553_si_001.pdf [file nz1c00553_si_001.pdf]

Energy vs. Charge Transfer in Manganese Doped Lead Halide Perovskites

Damiano Ricciarelli,[a,b] Daniele Meggiolaro,[b] Paola Belanzoni,[a,b] Asma A. Alothman,[c] Edoardo Mosconi,[b,c *] Filippo De Angelis[a,b,d*]

[a] *Department of Chemistry, Biology and Biotechnology, University of Perugia, Via Elce di Sotto 8, 06123 Perugia, Italy.*

[b] *Computational Laboratory for Hybrid/Organic Photovoltaics (CLHYO), Istituto CNR di Scienze e Tecnologie Chimiche "Giulio Natta" (CNR-SCITEC), Via Elce di Sotto 8, 06123 Perugia, Italy.*

[c] *Chemistry Department, College of Science, King Saud University, Riyadh 11451, Saudi Arabiad*

[d] *CompuNet, Istituto Italiano di Tecnologia, Via Morego 30, 16163 Genova, Italy.*

Email: edoardo@thch.unipg.it, filippo@thch.unipg.it

Supporting Information

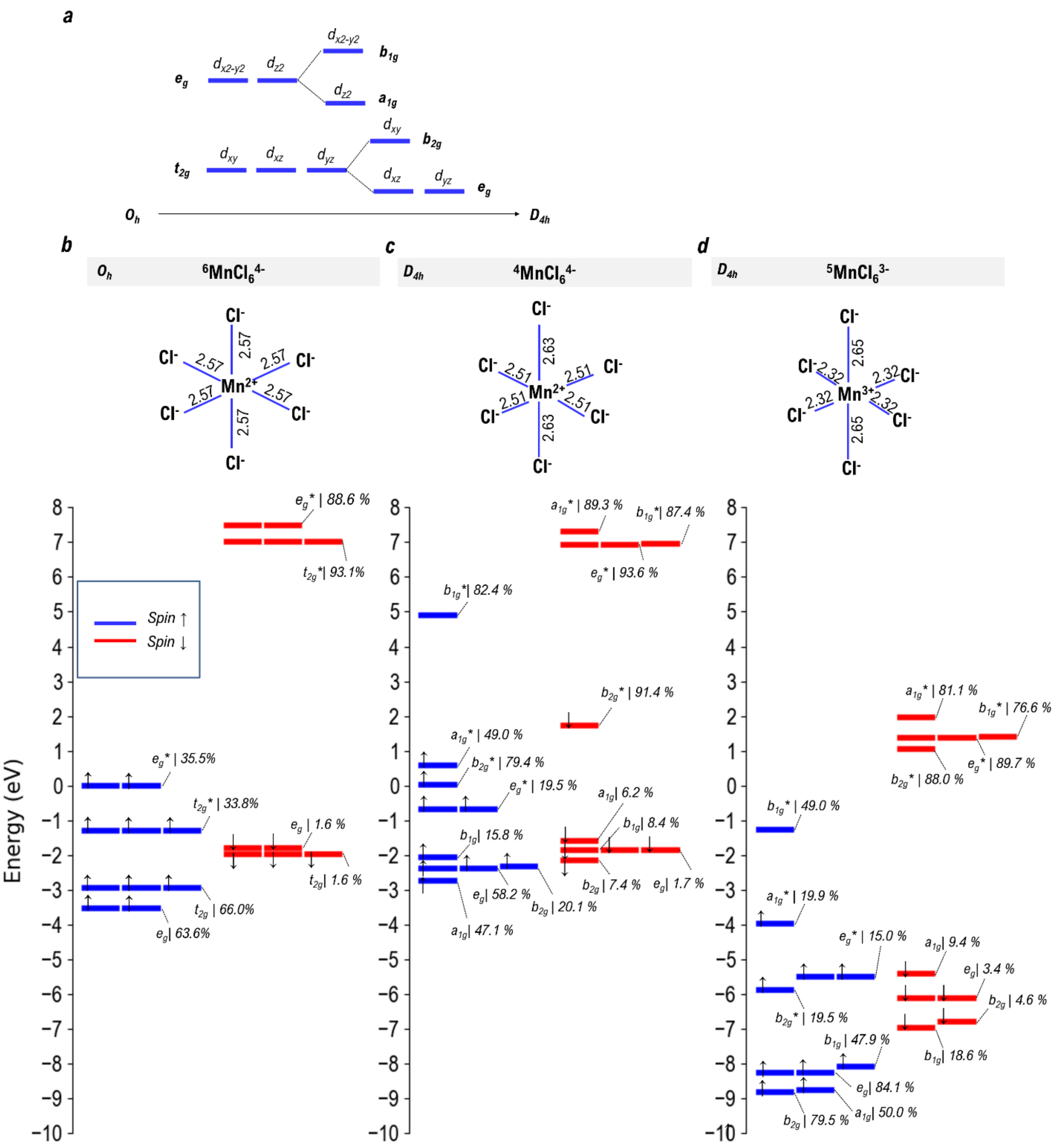

Figure S1. Electronic structure of the $MnCl_6^{4-}$ complex computed at the sextet, quartet and quintet/oxidized spin/charge states. a) Mn d orbital splitting when moving from an octahedral (O_h) symmetry to a tetragonally distorted (D_{4h}) symmetry. Employed models and molecular orbital diagrams with single particle states character and Mn percentages for b) $^6MnCl_6^{4-}$, c) $^4MnCl_6^{4-}$ and d) $^5MnCl_6^{3-}$. Energy values are referenced to $^6MnCl_6^{4-}$ HOMO.

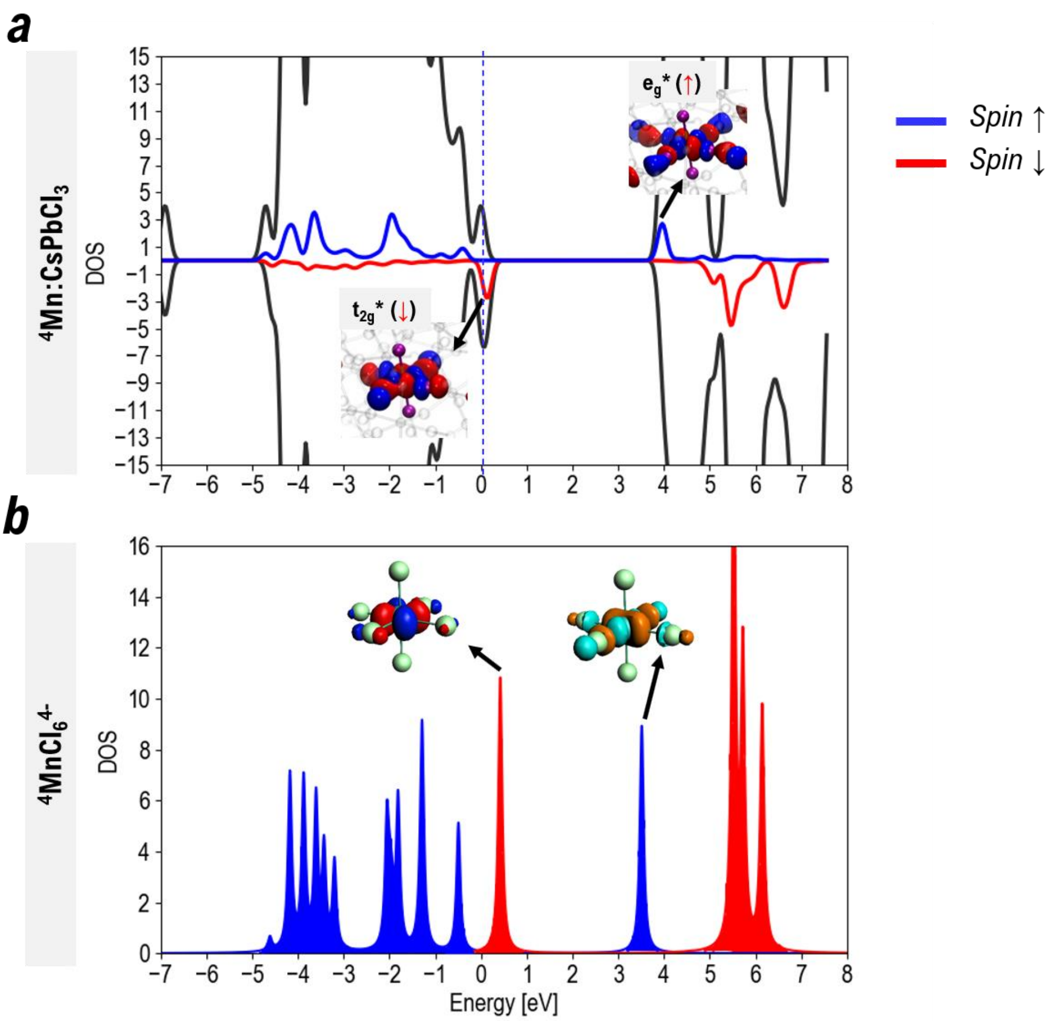

Figure S2 Comparison of DOS and boundary orbital between $^{4}Mn^{2+}:CsPbCl_3$ and $^{4}MnCl_6^{4-}$. a) DOS of $^{4}Mn^{2+}:CsPbCl_3$ computed at the PBE0 level of theory, the total contributions are coloured gray, while, the Mn ones are coloured in blue for the alpha manifold and in red for the beta manifold. The dashed bar represents the Fermi level. b) Mn d DOS of the model $^{4}MnCl_6^{4-}$ specie aligned to $^{4}Mn^{2+}:CsPbCl_3$ using the e_g peak. In the two figures the Isodensity plots of HOMO beta and LUMO alpha single particle states are reported for a qualitative comparison of their character between the solid and the complex.

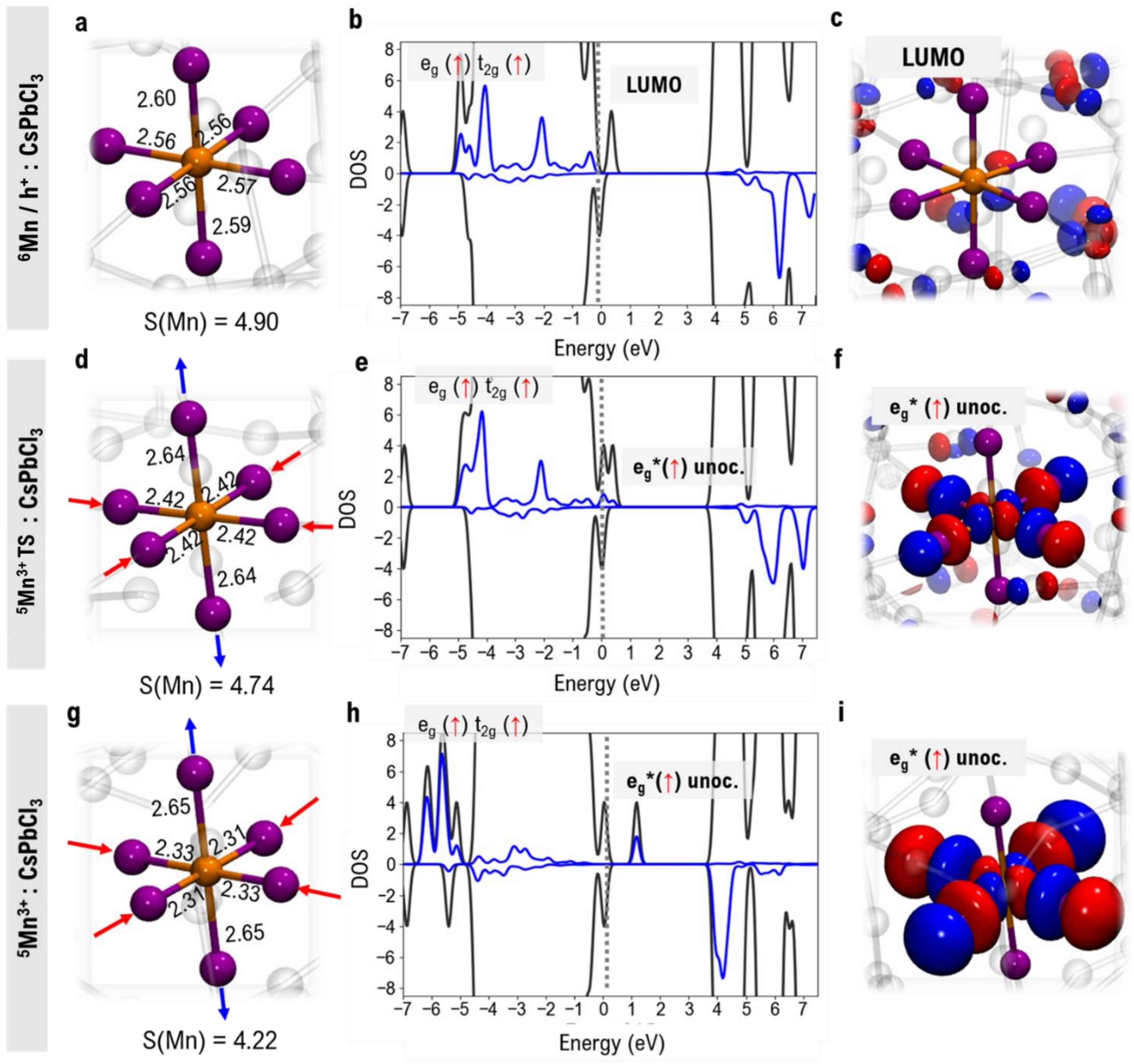

Figure S3. Structural and electronic properties of Mn:$CsPbCl_3$ in the quintet/oxidized spin state. a) $^6Mn^{2+}/h^+$:$CsPbCl_3$ first coordination sphere with distances in Angstrom and spin moments from Mulliken population analysis. Orange spheres represent Mn atoms, purple ones chloride atoms, species outside the Mn first coordination shell are transparent. b) Total (grey) and Manganese (blue) DOS (with negative/positive spin differentiation) for $^6Mn^{2+}/h^+$:$CsPbCl_3$ computed at the PBE0 level of theory with Mn molecular orbital labels. The dashed vertical bar represents the Fermi level. c) Isodensity plots of LUMO single-particle state. d) $^5Mn^{3+}$ TS:$CsPbCl_3$ first coordination sphere e) Total (grey) and Manganese (blue) DOS for $^5Mn^{3+}$ TS:$CsPbCl_3$. f) Isodensity plot of an unoccupied e_g* (↑) (LUMO) single particle state of $^5Mn^{3+}$ TS:$CsPbCl_3$.

g) $^{5}Mn^{3+}$:$CsPbCl_3$ first coordination sphere. h) Total (grey) and Manganese (blue) DOS for $^{5}Mn^{3+}$:$CsPbCl_3$.

i) Isodensity plot of an unoccupied e_g* (↑) (LUMO) single particle state of $^{5}Mn^{3+}$:$CsPbCl_3$.

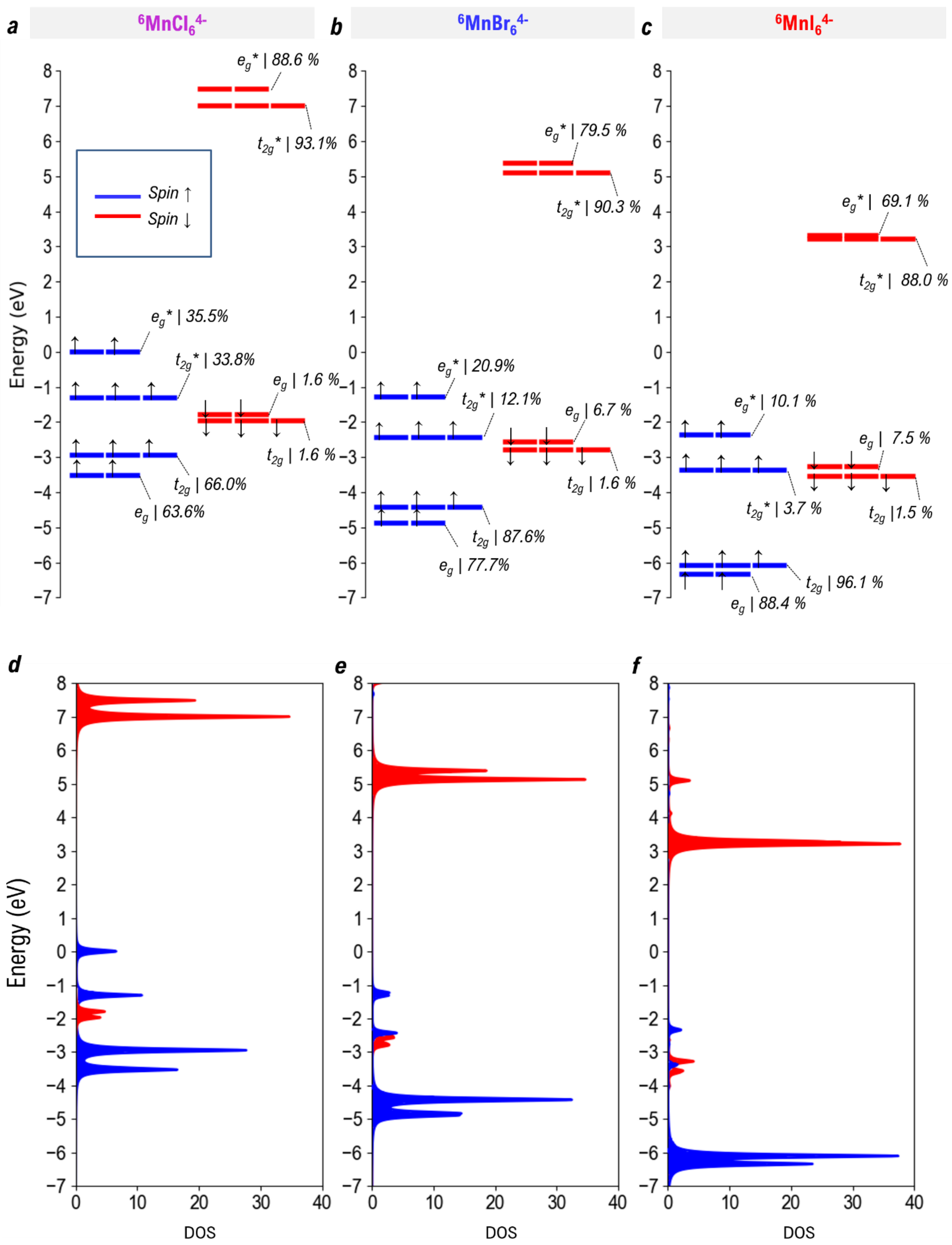

Figure S4. Molecular orbital diagram with single particle states characters and Mn percentages for a) $^{6}MnCl_6^{4-}$, b) $^{6}MnBr_6^{4-}$,c) $^{6}MnI_6^{4-}$ complexes. Mn contributions to DOS for d) $^{6}MnCl_6^{4-}$, e) $^{6}MnBr_6^{4-}$,f) $^{6}MnI_6^{4-}$ complexes. Energy values are referenced to $^{6}MnCl_6^{4-}$ HOMO.

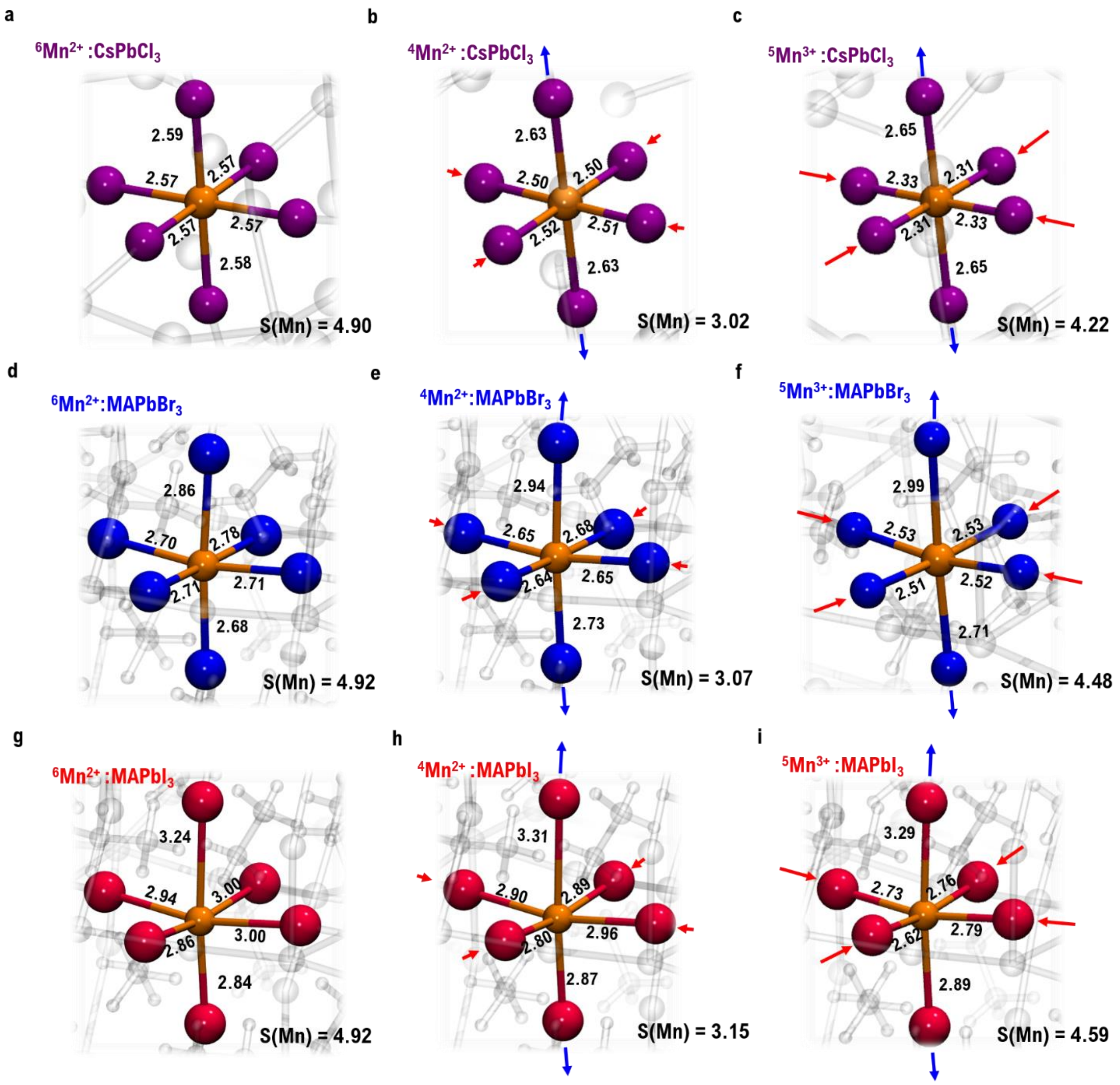

Figure S5. Structural features of Mn coordination sphere in different Cs(MA)PbX_3 materials for the respective sextet $^6Mn^{2+}$, quartet $^4Mn^{2+}$ and quintet/oxidized $^5Mn^{3+}$ states. Mn Spin Moments are reported along with octahedron distances in Angstrom. Orange spheres represent manganese atoms, purple ones chloride atoms, blue ones bromide atoms, red ones iodide atoms, species outside the Mn first coordination shell are transparent.

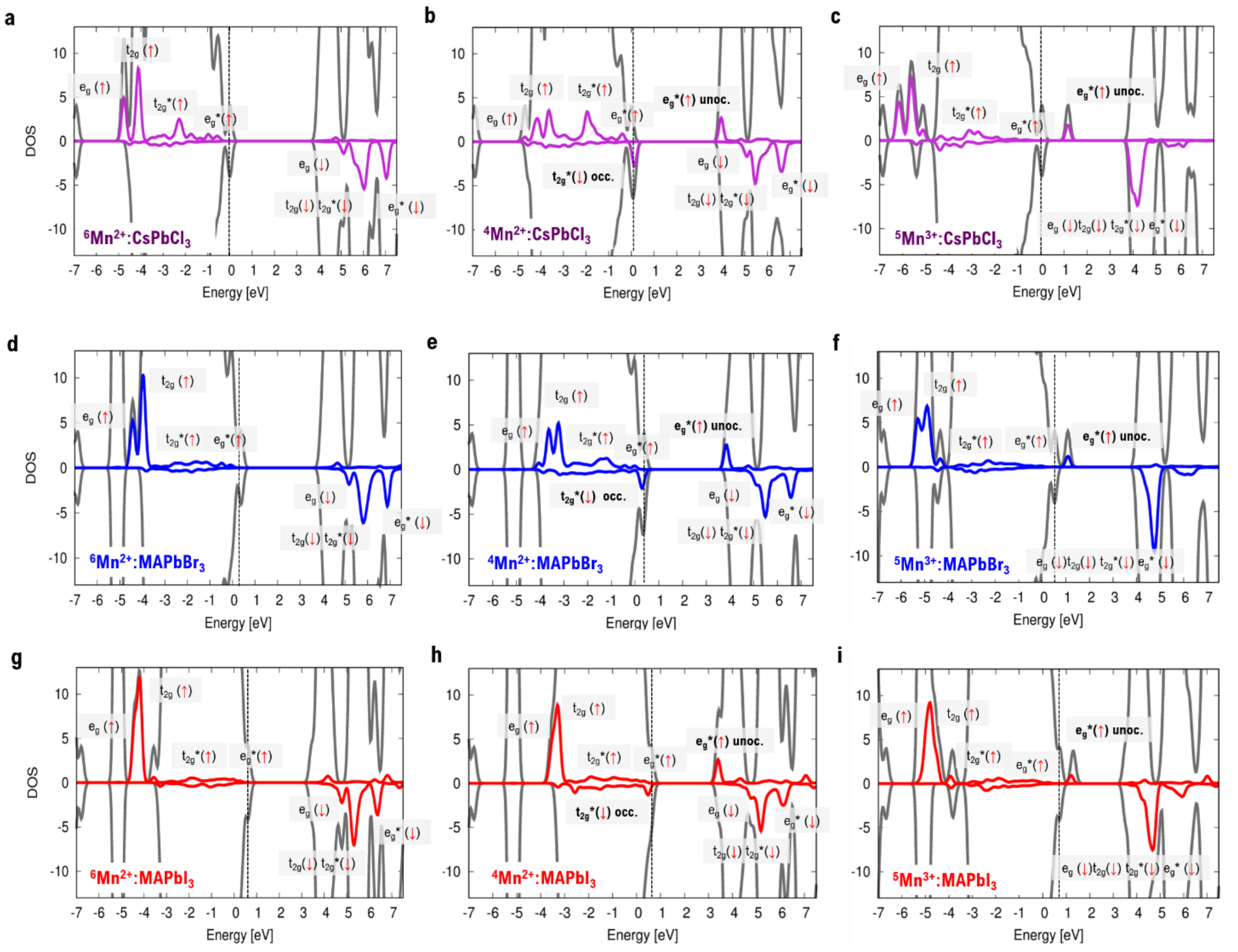

Figure S6. Total (grey) and Mn (colored) DOS (with positive/negative spin differentiation) for the Mn doped different Cs(MA)PbX_3 materials in the various spin states, i.e. sextet $^6Mn^{2+}$, quartet $^4Mn^{2+}$ and quintet/oxidized $^5Mn^{3+}$. The dashed bar represents the Fermi level. Purple lines represents Mn contribution in the chloride, blue ones Mn contribution in the bromide and red ones Mn contribution in the iodide.

Mn doublet higher energy state. In the case of the quartet Mn^{2+}:$MAPbI_3$ we obtained, additionally to $^4Mn^{2+}$:$MAPbI_3$ (1.99 eV above the ground state) a higher energy state, 5.25 eV above the $^6Mn^{2+}$:$MAPbI_3$ ground state, characterized by a calculated Mn spin moment of 1.12. The DOS in Figure S7 indicates that in this higher energy state a triplet VB-CB host is formed which is weakly coupled to a Mn-doublet with all (↑) t_{2g} and all (↓) e_g electrons, hence $^2Mn^{2+}$:$^3(MAPbI_3)$. A possible reason why we detect this species in iodide and not in the chloride/bromide may be ascribed to the reduced covalency and ligand strength of the Mn-X bond, represented by the B Racah parameter in d^5 Tanabe-Sugano diagram, which allows for the formation of a Mn doublet (t_{2g}^3 alpha and e_g^2 beta).[1]

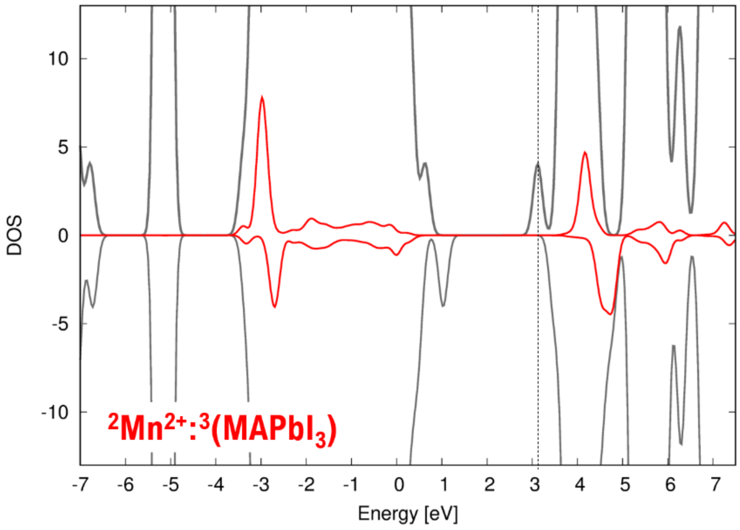

Figure S7. DOS of $^2Mn^{2+}$:$^3(MAPbI_3)$ with positive/negative spin differentiation. Total contributions are colored gray, Manganese ones red. The dashed bar represents the Fermi level.

References

(1) Li, W.-K. Matrix elements for configuration d5 in weak octahedral field using Racah methods. *Spectrochimica Acta Part A: Molecular Spectroscopy* **1968,** *24*, 1573-1579.

$Mn:CsPbCl_3$ properties at the PBE level of theory. We compute structures and quartet/sextet spin gaps at the PBE level of theory for $Mn:CsPbCl_3$. Concerning the sextet, we observe geometrical similarities between PBE and PBE0, as shown in Table S1 to be compared with Table 1, while PBE leads to a significant distortion at the Mn site for the quartet structure (not experienced in the case of PBE0). The PBE $^4T_1 \rightarrow ^6A_1$ spin transition, of ~ 1.5 eV, is lower than the hybrid (PBE0) one and it does not much the Mn luminescence recorded experimentally of ~ 2.0 eV.[1]

Table S1. Averaged Mn-X axial (d_{Mn-X} ax) and equatorial (d_{Mn-X} eq) distances (Å), Mn spin (e), band gaps (ΔE_{gap}, V) and $^4T_1 \rightarrow ^6A_1$ spin gaps ($\Delta E\ ^4T_1/^6A_1$, eV) computed at the PBE level of theory.

**$CsPbCl_3$**	**ΔE_{gap} =2.98**		**$\Delta E\ ^4T_1/^6A_1$ =1.48**
	d_{Mn-X} (ax)	d_{Mn-X} (eq)	Mn spin
$^6Mn^{2+}$	2.57	2.53	4.43
$^4Mn^{2+}$	2.39	2.72	2.80

Differences with PBE0 are retrieved also in the density of states (Figure S8). In the sextet we observe the e_g* peak to be aligned with the valence band of the material (Figure S8a) and, additionally, the repartition of the Mn states is inverted if compared to PBE0 with the bonding shell being more contributed by the halide and the anti-bonding one bearing more Mn content. In the quartet state (Figure S8b) we observe most of the Mn orbitals, previously located below the valence band, to emerge in the band gap of the material. On overall this analysis suggests the PBE functional gives rise to many differences in the description of the Mn doped material if compared with PBE0. We have noticed that the superiority of hybrid functionals over GGA ones in describing the electronic properties of Mn oxides has been reported in the literature.[2-5] In this framework, the bias of GGA may be ascribed to the Mn $3d^5$ shell destabilization provided by self-interaction, so one should prefer DFT+U, functionals containing HF exchange or GW approaches to simulate the

properties of Mn compounds. Based on all considerations pointed out in this paragraph, we decide to employ a PBE0 functional to carry out our study.

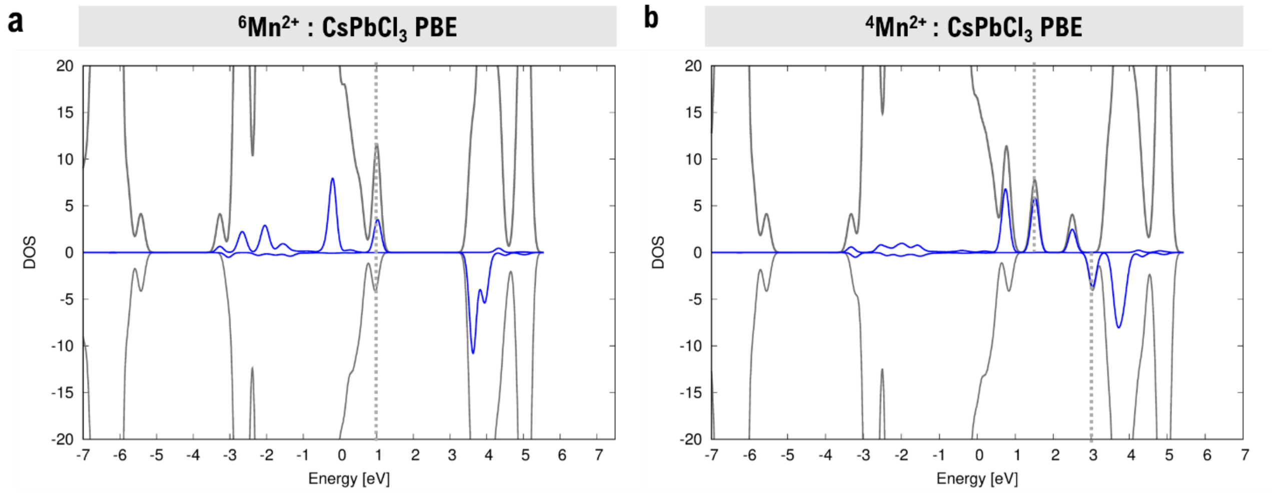

Figure S8. DOS of $^4Mn^{2+}$:$CsPbCl_3$ with positive/negative spin differentiation computed at the PBE level of theory. Total contributions are colored gray, Manganese ones blue. The dashed bar represents the Fermi level.

References

(1) Parobek, D.; Roman, B. J.; Dong, Y.; Jin, H.; Lee, E.; Sheldon, M.; Son, D. H. Exciton-to-Dopant Energy Transfer in Mn-Doped Cesium Lead Halide Perovskite Nanocrystals. *Nano Lett.* **2016,** *16*, 7376-7380.

(2) Tran, F.; Blaha, P.; Schwarz, K.; Novák, P. Hybrid exchange-correlation energy functionals for strongly correlated electrons: Applications to transition-metal monoxides. *Phys. Rev. B* **2006,** *74*, 155108.

(3) Stroppa, A.; Kresse, G.; Continenza, A. Revisiting Mn-doped Ge using the Heyd-Scuseria-Ernzerhof hybrid functional. *Phys. Rev. B* **2011,** *83*, 085201.

(4) Franchini, C.; Podloucky, R.; Paier, J.; Marsman, M.; Kresse, G. Ground-state properties of multivalent manganese oxides: Density functional and hybrid density functional calculations. *Phys. Rev. B* **2007,** *75*, 195128.

(5) Franchini, C.; Bayer, V.; Podloucky, R.; Paier, J.; Kresse, G. Density functional theory study of MnO by a hybrid functional approach. *Phys. Rev. B* **2005,** *72*, 045132.
